# Resonance behavior of embedded and freestanding microscale ferromagnets

**DOI:** 10.1038/s41598-022-15959-0

**Published:** 2022-08-31

**Authors:** Hamza Cansever, Md. Shadab Anwar, Sven Stienen, Kilian Lenz, Ryszard Narkowicz, Gregor Hlawacek, Kay Potzger, Olav Hellwig, Jürgen Fassbender, Jürgen Lindner, Rantej Bali

**Affiliations:** 1 Institute of Ion Beam Physics and Materials Research, Helmholtz-Zentrum Dresden-Rossendorf, Bautzner Landstraße 400, 01328 Dresden, Germany; 2 Institute of Solid State and Materials Physics, Technische Universität Dresden, 01069 Dresden, Germany; 3 Institute of Physics, Technische Universität Chemnitz, 09126 Chemnitz, Germany

**Keywords:** Applied physics, Materials for devices, Magnetic properties and materials

## Abstract

The ferromagnetic resonance of a disordered A2 Fe_60_Al_40_ ferromagnetic stripe, of dimensions 5 µm × 1 µm × 32 nm, has been observed in two vastly differing surroundings: in the first case, the ferromagnetic region was surrounded by ordered B2 Fe_60_Al_40_, and in the second case it was free standing, adhering only to the oxide substrate. The embedded ferromagnet possesses a periodic magnetic domain structure, which transforms to a single domain structure in the freestanding case. The two cases differ in their dynamic response, for instance, the resonance field for the uniform (*k* = 0) mode at ~ 14 GHz excitation displays a shift from 209 to 194 mT, respectively for the embedded and freestanding cases, with the external magnetic field applied along the long axis. The resonant behavior of a microscopic ferromagnet can thus be finely tailored via control of its near-interfacial surrounding.

## Introduction

Magnetic materials can prove vital for producing on-chip microwave elements such as antennas and spin-waveguides for GHz applications^1–13^. Further advances in microwave applications may be realized by rapidly prototyping and testing meso- and nano-scale magnetic elements for their resonant behavior under microwave excitations.

Local, nanoscale activation of ferromagnetism in nonferromagnetic templates can provide a powerful pathway to test the static and dynamic response of ferromagnets of desired geometric forms. The nonferromagnetic templates can consist of thin films possessing ordered lattice structures, wherein the activation of ferromagnetic regions can be achieved by local manipulation of the lattice structure, via focused ion or laser irradiation^14–17^. These ferromagnetic regions, realized by local lattice disordering, are surrounded by the ordered lattice of the nonferromagnetic template material. The surrounding material can influence the magnetic properties of the embedded structures. Knowledge of how the nonferromagnetic surrounding influences the response of embedded structures is essential for applications, however a direct comparison has so far been difficult to achieve. A direct writing approach could be used for sensitive tuning of the resonant behavior by controlling the geometry of the embedded ferromagnetic objects.

The realization of an embedded ferromagnet and the comparison with the same structure in freestanding form was achieved using B2-ordered Fe_60_Al_40_ films as templates. The Fe and Al atoms can be forced to swap their site-occupancies that determine the B2 order and form the disordered A2 structure, which is ferromagnetic. The site-swapping process can be realized using a highly focused beam of noble-gas ions^18^ or local laser melting followed by rapid solidification^19^ in a stylus-like fashion. The transition from the B2 to A2 structure results in an increase in the Fe–Fe nearest-neighbor interactions as well^20–22^ as a ~ 1% expansion of the unit-cell volume^23–26^. In an A2 lattice formed within a B2 lattice surrounding, the volume expansion can be anisotropic, depending on the aspect ratio of the disordered region. In particular, in high-aspect-ratio objects, such as the ferromagnetic structures considered here, the lattice expansion along the long edge can be lower than the expansion along the short edge^14^. This lattice anisotropy may induce observable magnetic effects, which we seek to investigate.

The B2 ordered material surrounding the embedded ferromagnetic region consisting of A2 Fe_60_Al_40_ can be removed using again a focused ion beam with parameters designed for etching rather than lattice disordering, thereby releasing the anisotropic strain in the irradiated region. Microresonators allow for ferromagnetic resonance (µFMR) measurements and a comparison of identical embedded versus freestanding magnetic elements, thus comprising a powerful approach for the rapid prototyping of magnetic devices of desired nanoscale geometries. A variety of magnetically writeable alloys are available, such as B2 Fe_50_Rh_50_^27,28^, Fe_60_V_40_, etc.^29,30^, thereby opening vast possibilities for achieving magnetic elements with designed resonance behavior, depending on the selection of the template material as well as the nanomagnet shape.

Here we perform a direct comparison of the magnetic properties of an embedded ferromagnetic structure within a paramagnetic template, to that of the same structure in freestanding form, achieved by removal of the surrounding material. The template material deployed here is 32 nm thin B2 Fe_60_Al_40_.

## Methods

**Figure 1:**
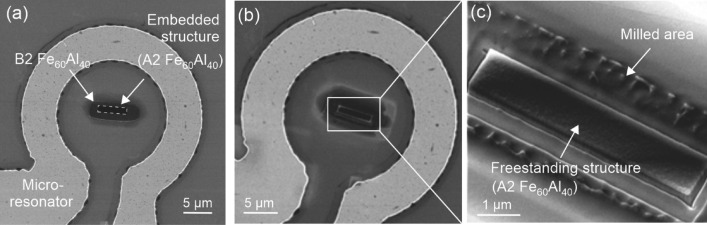
(**a**) HIM image of the A2 Fe_60_Al_40_ strip embedded in a B2 Fe_60_Al_40_ template (**b**) Milling of the region around the A2 Fe_60_Al_40_ strip to produce a freestanding structure, detailed in (**c**).

Fe_60_Al_40_ films of thickness 32 ± 2 nm were grown on SiO_2_/Si substrate by magnetron sputtering from a target of the same composition, with the substrate held at 300 K. To achieve the B2 ordering, the films were annealed at 773 K in vacuum. For the microresonator experiment, templates of 10 µm × 4 µm dimensions were prepared in the B2 Fe_60_Al_40_ using photolithography techniques, followed by the removal of exposed SiO_2_ by etching. The removal of the SiO_2_ is necessary in order to avoid degradation of the microresonator`s performance due to increased loss, caused by an induced charge layer under the SiO_2_/Si interface^31^. In the next lithography step, an Ω-shaped microresonator structure, with an inner diameter of 20 µm was patterned around the B2 Fe_60_Al_40_ template (Fig. 1). The microresonator stack consists of 5 nm Cr/600 nm Cu/100 nm Au, prepared by e-beam evaporation. The reverse side of the chip was metallized with 5 nm Cr/300 nm Cu/100 nm Au to form a ground plane of the microstrip line and provide a microwave current return path. Details on the film growth and properties of Fe_60_Al_40_ films can be found in Ref.^14^ and on the microresonator method in Refs.^6,13^.

The B2 Fe_60_Al_40_ template positioned at the centre of the microresonator acts as a testbed for prototyping meso- and nano-scale magnets. The ferromagnetic structure of 5 μm × 1 μm area has been written into the template film using a ~ 2-nm-diameter Ne^+^ ion beam with an energy of 25 keV and a fluence of 10 ions/nm^2^. In that way, the maximum *M*_*s*_ of 708 kA/m was achieved in the irradiated region^10^. A Carl Zeiss Orion NanoFAB helium ion microscope with a NPVE pattern generator from Fibics has been employed for this task^32^. To minimize collateral ion beam damage imaging with He and Ne has been reduced to a minimum and no high resolution images have been recorded. In Fig. 1 the boundary of the irradiated area is indicated by a dashed line. To compare the properties of the embedded ferromagnet with that of a freestanding magnet from the same template, the material outside of the border was milled away using the Ne^+^ ion beam. Milling has been performed at a Ne^+^ energy of 15 keV and a fluence of 7500 ions/nm^2^ for rough milling. The rough milling step was followed by a polishing step with 5000 ions/nm^2^ for achieving smooth edges of the freestanding magnet (Fig. 1b,c). After the milling process, the previous irradiation treatment to produce a magnetized region viz., irradiation with 25 keV Ne^+^-ions at a fluence of 10 ions/nm^2^ was repeated on the freestanding region to ensure that the Fe and Al site-occupancies remain in a fully randomized state, giving the maximum magnetization.

In addition to the microresonator FMR, characterization of the embedded and freestanding structures has been performed using atomic force microscopy (AFM) as well as magnetic force microscopy (MFM) in lift mode, with a Bruker Dimension Icon (ScanAsyst) microscope at a lift height of 30 nm using a CoCr coated tip. Micromagnetic simulations were performed using the open source Mumax^3^ code^33^. In particular, the continuous wave FMR method was used^34^. To simulate the FMR spectra, additional anisotropies, i.e., the uniaxial in-plane anisotropies *K*_*2||*_ and the out-of-plane anisotropy *K*_*2⊥*_, were included in the simulations. To obtain a convergent solution the effect of the paramagnetic surrounding on the embedded ferromagnet has been simulated by varying the two anisotropy terms. Exemplary simulation results can be seen in the Supplementary Information.

## Results and discussions

**Figure 2:**
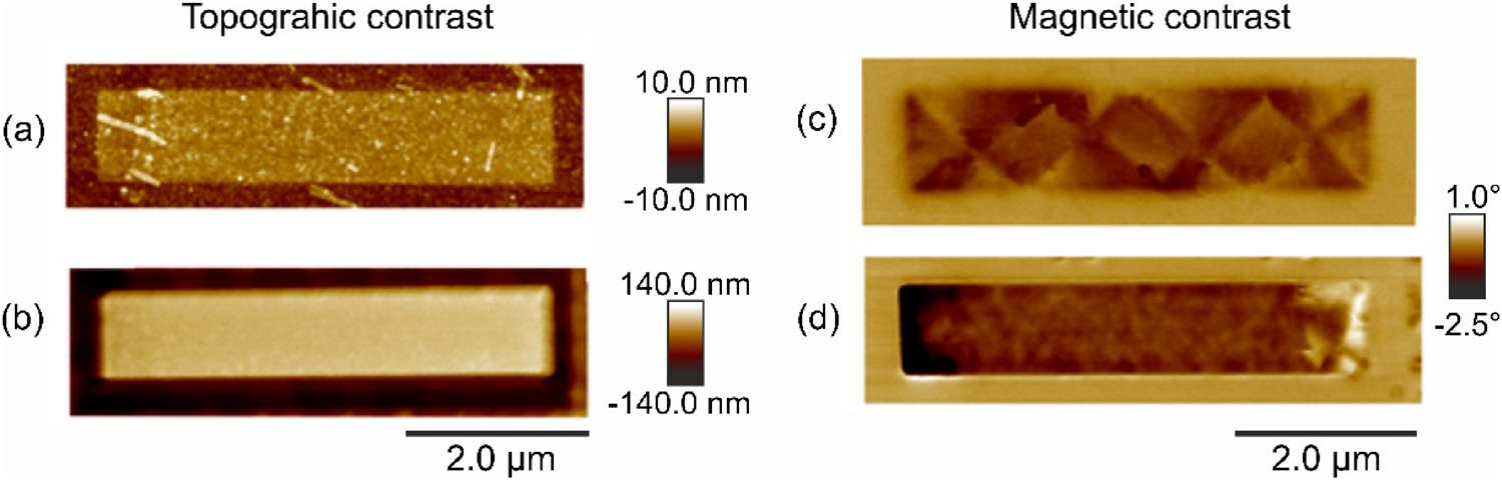
(Color online) Atomic force microscopy images (**a**) of the region irradiated with a highly focused Ne^+^-ion beam at 25 keV and a fluence of 10 ions/cm^2^ thereby forming disordered A2 Fe_60_Al_40_ embedded within a B2 Fe_60_Al_40_ surrounding. (**b**) The same region as in (**a**) after milling away the B2 Fe_60_Al_40_ surrounding. (**c**, **d**) Magnetic force microscopy images showing the magnetic phase contrast corresponding to the topographic contrast in (**a**) and (**b**), respectively.

The magnetic force micrographs along with the topography, for the embedded and freestanding cases are shown in Fig. 2. In the topographic contrast the embedded ferromagnet (Fig. 2a), shows an increased height of ~ 2 nm at the ion-irradiated region. After the milling process, the structure is surrounded by a trench of ~ 250 nm depth, i.e., it is freestanding on the SiO_2_ surface (Fig. 2b). The dimensions of the structure are largely maintained during the milling process. The length and width of the embedded structure are 5 and 1 µm respectively, whereas for the freestanding structure, the dimensions are 5 and 0.92 µm, respectively. Although the diameter of the Ne^+^-beam used for milling is ~ 2 nm, lateral ion scattering can extend ~ 100 nm, lowering the resolution of the milling process. In addition, artefacts such as drift resulted in the width of the milled structure being 80 nm less than that of the embedded structure.

Significant differences between the magnetic domain structures of the embedded and freestanding cases are observed in MFM^14^. The embedded ferromagnet possesses a periodic magnetic in-plane domain pattern, with a periodicity of ~ 1 µm. At the two ends of the structure, the pattern resembles Landau domains typically observed in square shaped ferromagnetic objects. The Landau-like domains appear to propagate along the long-axis of the structure, thus defining the domain periodicity of ~ 1 µm as seen in Fig. 2c. In contrast, the freestanding ferromagnet shows low magnetic contrast along the long-axis. The magnetic poles are located at the two far ends of the freestanding structure, where the stray fields are concentrated, as indicated by the bright and dark contrast seen in Fig. 2d. This suggests a single-domain state with the magnetization lying in-plane along the long-axis of the structure, as it is typical in the case of a high-aspect-ratio geometry. Perturbations of the domain state can occur, as seen in Fig. 2d, presumably due to an increased roughness at the edges of the freestranding stripe, caused by the milling process. In comparison, the embedded structure is expected to possess smooth edges due to the lower ion-fluences used to generate the ferromagnetic stripe.

**Figure 3:**
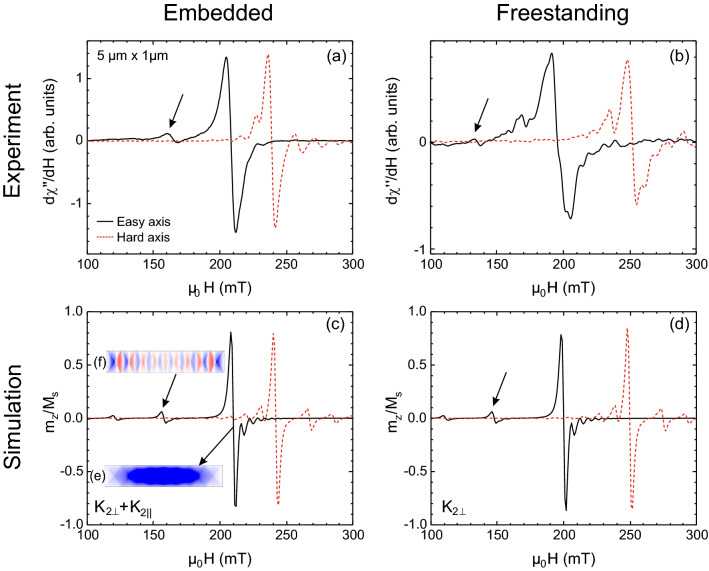
(Color online) FMR spectra observed at a frequency of 13.9 GHz, (**a**) for the A2 Fe_60_Al_40_ ferromagnet surrounded by B2 Fe_60_Al_40_ and (**b**) for the freestanding A2 Fe_60_Al_40_ ferromagnet. (**c**, **d**) show the corresponding micromagnetic simulations of the embedded and freestanding sample, respectively. Measurements/simulations with the external field along the magnetically easy (hard) axis are shown by black solid (red dotted) lines, respectively. The insets show snapshots of (**e**) the quasi uniform (center) mode and (**f**) a standing spin wave mode using a red-white-blue colour scale, where the magnitude of the transient magnetization *z*-component, ± *m*_*z*_; white indicates *m*_z_ = 0 and red (blue) regions possessing + (−) *m*_z_.

The dynamic response of the ferromagnet for the embedded and freestanding cases has been measured by FMR at an excitation frequency of 13.9 GHz. For each sample measurements were taken with the static external magnetic field applied in-plane along the long axis as well as along the short axis. These directions correspond to the magnetically easy axis (EA) and hard axis (HA) in accordance to the shape anisotropy considerations for this high-aspect ratio. As seen in Fig. 3, well-defined absorption lines are observed in both cases. Each resonance line corresponds to a certain precession mode within the ferromagnetic region. Here we first focus on the main absorption (Kittel mode) line, which is known to correspond to the *k* = *0*, i.e. the uniform precession mode. The resonance fields *H*_*res*_, at which the *k* = *0*, uniform resonant absorption occurs, have been tabulated in Table 1 for the embedded as well as the freestanding structures for the EA as well as the HA field directions.

**Table 1:** Comparison of the uniform resonance modes obtained from experiments and simulations for the embedded and freestanding structures for both easy and hard axis geometry.

	Embedded structure				Freestanding structure			
	*µ*_*0*_*H*_res_ (mT)		*K*_*2*_ (kJ/m^3^)		*µ*_*0*_*H*_res_ (mT)		*K*_*2*_ (kJ/m^3^)	
	Easy axis	Hard axis	*K*_2⊥_	*K*_2ǁ_	Easy axis	Hard axis	*K*_2⊥_	*K*_2ǁ_
Measurement	209	239	–	–	194	252	–	–
Simulation	210	242	30	3.5	200	250	30	0

As seen in Fig. 3a and b, distinct shifts of the resonance fields are observed, depending on whether the ferromagnet is embedded or freestanding. For instance, the resonances with the field parallel to the EA are at 209 mT and 194 mT for the embedded and freestanding case, respectively. Conversely, with the field applied parallel to the HA, the resonance of the freestanding structure occurs at 252 mT, whereas it is at 239 mT for the embedded structure.

Whereas the anisotropy constants, driven by the strain, play a key role in determining the position of the resonance lines, the linewidths are determined by dynamic effects, in particular damping. A detailed study of damping mechanisms is beyond the scope of this work, nevertheless differences in the linewidths are clearly observed. The embedded ferromagnet exhibits a linewidth *µ*_*0*_*ΔH*_*pp*_ of 6.9 and 5.4 mT for the easy and hard axis, respectively. For the freestanding case, the corresponding linewidths are 5.9 and 7.4 mT, respectively. These linewidth values have been estimated using lorentzian fitting (see Supplementary Information). We note that the linewidths of the freestanding structure are smaller with the field along the EA in comparison to the HA, whereas the trend is inverted for the embedded structure, where it is the EA along which the larger linewidth is observed.

Micromagnetic simulations using Mumax3 were used to replicate the experimental results and obtain insight into the reasons for the differences in the embedded and freestanding ferromagnet. The material surrounding the embedded magnet is paramagnetic and cannot be implemented directly into the present simulation, since a converging solution is not reached for the paramagnetic environment. Therefore the role of the paramagnetic surrounding in this case has been investigated in terms of anisotropy constants that determine the FMR line position. Dynamic effects from any potential distortions of the stray field due to the presence of the paramagnetic surrounding should appear in the linewidths, however these will not be considered in the above simulation.

A high-aspect-ratio ferromagnetic structure can be expected to possess a dominant shape anisotropy term, favoring a moment orientation in the plane parallel to the long axis. The micromagnetic simulation implicitly considers the shape anisotropy term due to the dimensions of the sample. In addition, further anisotropy terms are explicitly included in the calculation, to reproduce the observed FMR spectra. Two uniaxial magnetic anisotropy components will be used to simulate the behavior of the ferromagnets; the anisotropy component- *K*_2⊥_, favoring an out-of-plane moment orientation (which is related to surface anisotropy) and an in-plane anisotropy component- *K*_2||_, favoring a moment orientation parallel to the short axis of the ferromagnetic structures. The *K*_2⊥_ term has been previously observed in uniformly irradiated continuous thin films, and attributed to interfacial roughness^38^. Conversely, the *K*_2ǁ_ term has been attributed to compressive strains due to difference in the lattice parameter of the matrix surrounding the embedded structure^14^. The presence of *K*_2ǁ_ has been shown to drive the periodic domain pattern seen in Fig. 2c. Since the periodic domains are absent in the freestanding structure, the *K*_2ǁ_ can be neglected in the latter case.

In the simulation, the following magnetic parameters are used: saturation magnetization *M*_*s*_ = 708 kA/m, exchange stiffness *A* = 4 pJ/m, *g-*factor = 2.087, damping constant *α* = 0.005, excitation frequency *f* = 13.9 GHz. The *A* has been obtained from Brillouin Light Scattering^35^ whereas *M*_*s*_ was obtained from magnetometry using a SQUID-VSM, in both cases on uniformly irradiated continuous thin films (not shown here). A rectangular shape with dimensions of 5 × 0.918 µm^2^ and 34 nm thickness was considered. This shape was divided into 1024 × 128 × 1 cells to optimize the speed vs. accuracy of the simulations.

The simulation results are shown in Figs. 3c and d, respectively for the embedded and freestanding cases. The absorption is simulated as the ratio of the z-component of the dynamic magnetization *m*_*z*_ normalized to *M*_*s*_^36^. The inset figures, Figs. 3e and f, show snapshots of the dynamic local magnetization for two selected modes, i.e., the main absorption line that typically corresponds to the uniform precession mode and a lower-field spin-wave mode. The red–white–blue color scale denotes the local out-of-plane component of the dynamic magnetization ± *m*_*z*_.

A variation of *K*_2⊥_ was performed to select a good fit to the experimentally observed resonance fields for the freestanding case, and finally *K*_2⊥_ = 30 kJ/m^3^ is selected. For the freestanding case, the input of *K*_2⊥_ alone is sufficient to obtain a good match with the experiment (Fig. 3). However, in the embedded case, in addition to *K*_2⊥_ = 30 kJ/m^3^ a *K*_*2||*_ of 3.5 kJ/m^3^ must be included to match the experiment. Table 1 lists the experimentally observed resonance fields as well as the simulated parameters for comparison.

The above results show that the resonance-field shift of the embedded ferromagnet, as compared to the freestanding structure, can be simulated via the addition of a common *K*_2⊥_ and a *K*_*2||*_ that is specific to the embedded case.

To understand the origin of *K*_2⊥_ and a *K*_*2||*_, it is necessary to consider the changes to the lattice caused by the irradiation-induced disordering. The Ne^+^-beam disorders the B2 lattice to form the A2 structure, where the latter possesses a ~ 1% larger unit cell by volume. The expansion of the A2 structure does not occur isotropically in space but tends to depend on the size of the disordered region.

In uniformly ion-irradiated, disordered Fe_60_Al_40_ thin films, the lattice expansion can be expected to occur largely along the z-axis, i.e. perpendicular to the film plane^37^. The perpendicular anisotropy component appears to be a feature of the disordered Fe_60_Al_40_, since it has been observed in disordered continuous thin films as well^38^. This suggests that in the present microscopic magnetic structures as well as in uniform thin films, a lattice relaxation along the z-axis occurs. The *K*_2⊥_ of 3 × 10^4^ J/m^3^ is much smaller than the shape anisotropy (4.8 × 10^7^ J/m^3^) and, therefore, does not significantly influence the static properties, such as the domain pattern. Consistent with the previous observation of *K*_2⊥_ in uniformly irradiated thin films, the above *K*_2⊥_ is necessary in the fitting of the line-positions, for both the embedded as well as the freestanding structures. The influence of the *K*_2⊥_ is nevertheless detectable in the resonance line positions under FMR via the microresonator.

Conversely, *K*_*2||*_ occurs only in the embedded ferromagnet and can be attributed to an anisotropic lattice expansion due to the high-aspect shape. This geometry-dependent structural anisotropy has been observed in Fe_60_Al_40_ microstructures where the lattice parameter along the short axis tends to be larger than the lattice parameter measured along the long-axis^14^. In embedded disordered regions, the laterally surrounding B2 lattice exerts a stress on the expanding A2 region. The preferential expansion observed along the short axis, is consistent with the occurrence of *K*_*2||*_, and the observation of the periodic domain structure in the embedded ferromagnet (Fig. 2a) as well as the corresponding shift in the *µ*_*0*_*H*_*res*_ (Fig. 3a). Furthermore, through a direct comparison, we find that the *K*_*2||*_ is a feature specific to embedded magnetic structures of meso- and nanoscopic dimensions.

Strain induced magnetic anisotropies have been exploited in literature to drive moments to orient along desired axes, whereby lattice mismatched substrates have been widely used^39–41^. The results above demonstrate an alternative route to induce magnetic anisotropies viz. by embedding ferromagnetic meso- and nanostructures within nonferromagnetic surroundings. The results in Fig. 3 and Table 1 show the effect of the surrounding material on the dynamic properties of the embedded sample, as a distinct shift in the resonance fields.

The main FMR absorption line originates from the uniform precession. This can be seen from the inset of Fig. 3e showing a snapshot of *m*_*z*_ having a homogeneous blue tint throughout the central region of the sample. The magnetization around the edges deviates from the uniform precession due to the presence of demagnetizing fields. In Fig. 3e, a periodic, vortex-like pattern in the simulated *m*_*z*_ occurs due to the interference of spin waves reflected from the assumed perfect surfaces of the structure. This pattern persists in the simulations, as the transient magnetization preocesses about the applied external field. In the real case however, the periodic pattern in the dynamic *m*_*z*_ vanishes due to imperfect reflections.

Compared to that, the selected lower-field mode at *µ*_*0*_*H*_*res*_ = 156 mT (see Fig. 3f) displays a standing spin-wave-mode pattern of alternating + *m*_*z*_ and -*m*_*z*_ color along the long axis. It is known from literature that modes occurring at fields lower than those of the uniform (*k *= 0) mode correspond to standing spin-wave modes, where the nonzero *k* is perpendicular to the magnetization^42^. This type of mode is expected to be sensitive to reflection and interference effects at the interfacial regions of the microstructure. The occurrence of this low-field mode is an indication that the magnetic interfaces of the embedded structure are at least as well defined, as those of the freestanding structure.

The microresonator FMR is sensitive to several other excitation modes observable as satellite lines in the spectra of Fig. 3. These absorption lines can be attributed to complex higher order spin-wave modes, such as localized modes^13^. Whereas a detailed study of these modes is beyond the scope of this work, we note that irradiation-assisted mesoscale ferromagnets possess sufficient smooth surfaces for the observation of nonuniform localized modes.

The occurrence of a high-permeability environment is likely to distort the magnetic stray fields around the embedded ferromagnet. However, the resonance shift observed here can be simulated purely within the framework of strain-related anisotropy terms. Despite neglecting the paramagnetic moments in the surrounding of the ferromagnetic area, i.e., employing strain induced additional anisotropy only, good agreement between the experimental data and the simulation to the FMR line position has been achieved.

## Conclusions

Observations of ferromagnetic resonance on a ferromagnetic stripe surrounded by non-ferromagnetic material is directly compared to its freestanding counterpart, showing a distinct shift of the resonance line. The microscopic ferromagnetic stripe is directly written onto a nonferromagnetic B2 Fe_60_Al_40_ template film via local disordering using a focused Ne^+^-beam. The resulting ferromagnetic structure is embedded within the nonferromagnetic B2 ordered surrounding material. A direct comparison of the embedded structure to a freestanding ferromagnetic stripe of similar dimensions, is achieved by readjusting the Ne^+^-beam parameters and completely milling away the B2 ordered surrounding, thus obtaining a freestanding ferromagnetic structure. The quasi-uniform resonant mode of the freestanding ferromagnet shows a shift towards lower fields in comparison to the resonance line of the embedded structure. The resonance shift is a signature of strain-induced anisotropies present in the embedded ferromagnet due to the B2 ordered surrounding. Therefore, the selective material removal can provide another pathway for the local modification of resonant properties, in addition to the local disordering of the B2 Fe_60_Al_40_ template material itself. Indeed, embedded ferromagnetic regions have recently been obtained on other template materials^43–46^. An amplification of stray field effects can be expected in dipolarly coupled objects, since the coupling is reliant entirely on the stray field alone. The above demonstration of rapid writing and detection of a single magnetic region can be advanced to coupled magnetic regions, where the selective removal of the intra-magnetic regions can result in tuning the collective resonance. This study provides a step towards applying embedded ferromagnetic meso- and nanostructures for devices where precise control of the microwave response is an important requirement.

## Supplementary Information

Supplementary Information.

## Data Availability

All data generated or analysed during this study are included in this published article (and its Supplementary Information files).
