# Supplementary material for: Resonance behavior of embedded and freestanding microscale ferromagnets
Source: Sci Rep. 2022 Aug 31;12:14809. doi: 10.1038/s41598-022-15959-0 (PMC9433406; doi:10.1038/s41598-022-15959-0)
Supplement: Supplementary file 1 — Supplementary Information. [file 41598_2022_15959_MOESM1_ESM.pdf]

Supporting Information

Resonance behavior of embedded and freestanding microscale ferromagnets

Hamza Cansever[1,*], Md. Shadab Anwar[1, 2], Sven Stienen[1], Kilian Lenz[1], Ryszard Narkowicz[1], Gregor Hlawacek[1], Kay Potzger[1], Olav Hellwig[1,3], Jürgen Fassbender[1,2], Jürgen Lindner[1], Rantej Bali [1,*]

[1]Helmholtz-Zentrum Dresden-Rossendorf, Institute of Ion Beam Physics and Materials Research, Bautzner Landstraße 400, 01328 Dresden, Germany
[2]Technische Universität Dresden, Institute of Solid-State Physics, 01069 Dresden, Germany
[3]Technische Universität Chemnitz, Institute of Physics, 09126 Chemnitz, Germany
** Corresponding authors*

Micromagnetic simulations were performed using open source Mumax³ code, in particular, the continuous wave FMR method was used [1], which is based on resonance equation [2] as follows;

$$(\omega/\gamma)^2 = \left[H_{res}\cos(\varphi-\varphi_H) - \frac{2K_{2\parallel}}{M_s}\right] \times \left[H_{res}\cos(\varphi-\varphi_H) + 4\pi M_{eff}\right] \qquad \text{(S1)}$$

Here is $\gamma = g \cdot \mu_B/\hbar$ denotes the gyromagnetic ratio, φ is the in-plane angle of the magnetization **M**, φ_H is the in-plane angle of the external magnetic field. $K_{2\|}/M_s$ is the uniaxial in-plane anisotropy, which was introduced to mimic the uniaxial symmetry of the rectangular geometry. The effective magnetization is defined as the difference between the saturation magnetization and the perpendicular uniaxial anisotropy field: $4\pi M_{eff} = 4\pi M_s - 2K_{2\perp}/M_s$.

For the simulations, the following magnetic parameters were used; saturation magnetization M_s = 708 kA/m, exchange stiffness A = 4 pJ/m, Landé g-factor = 2.087, Gilbert damping constant α = 0.005, which were obtained from an extended $Fe_{60}Al_{40}$ Ne^+-irradiated sample (not shown here) by FMR. The microwave frequency was set to 13.9 GHz. A rectangular magnetic strip with the dimensions of 5 × 0.918 µm² and 34 nm of thickness was considered. This shape was divided into 1024 × 128 × 1 cells to optimize the speed and the accuracy of the simulations. Additional to the shape anisotropy, we added a uniaxial out-of-plane

anisotropy, $K_{2\perp}$, to the simulation with varying thickness and $K_{2\perp}$ (1×10^3 J/m³, ..., 5×10^4 J/m³) for the easy axis (Fig S1) and hard axis (Fig S2) geometry.

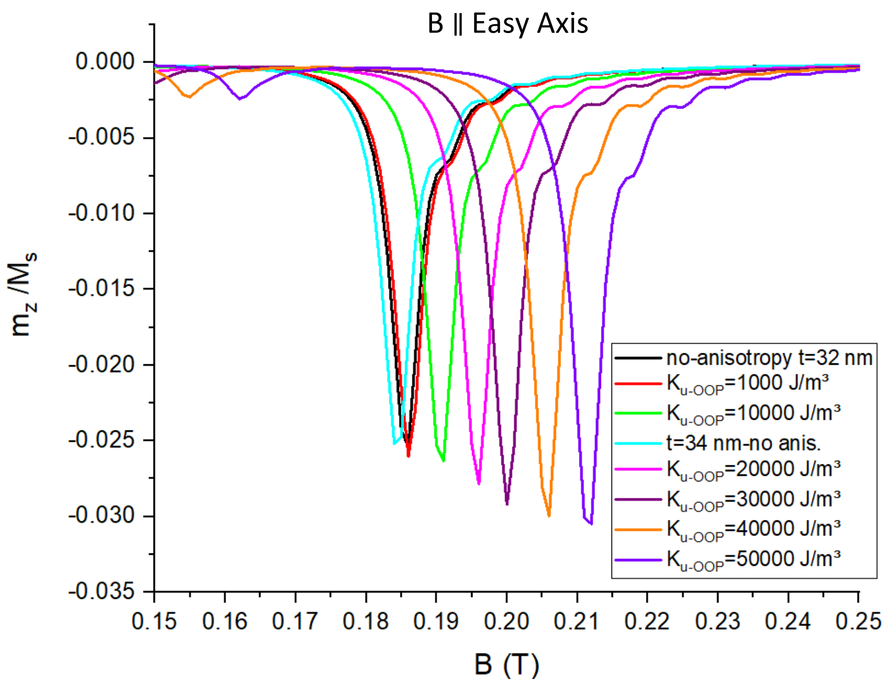

FIG S1. Micromagnetic simulations of the freestanding FM strip at 13.9 GHz with varying thickness and uniaxial out-of plane anisotropy $K_{2\perp}$. The external magnetic field was oriented parallel to the easy axis.

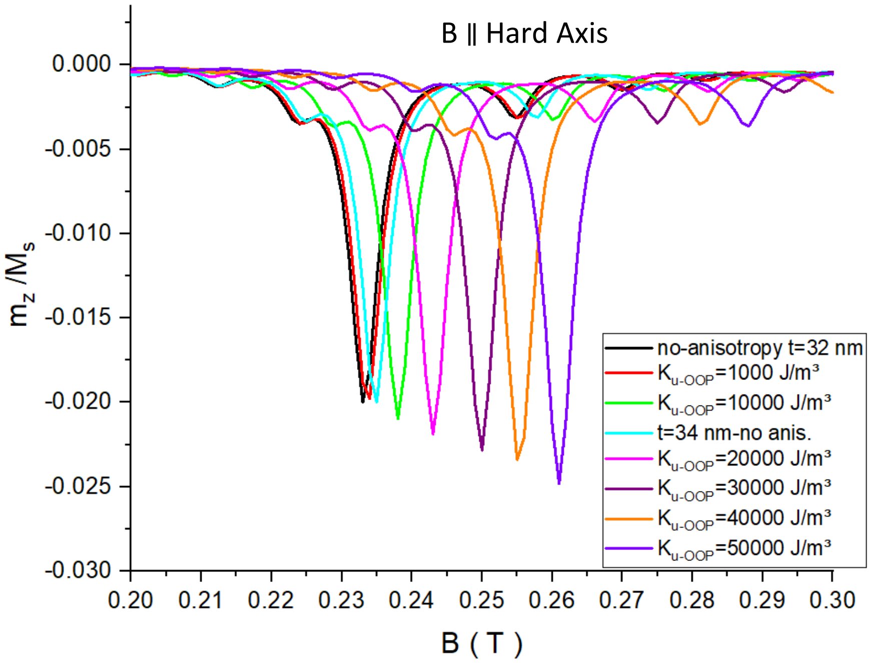

FIG S2. Micromagnetic simulations of the freestanding FM strip at 13.9 GHz for varying thickness and uniaxial out-of plane anisotropy, with the external field oriented parallel to the hard axis.

Lorentzian fits to evaluate the FMR spectra:

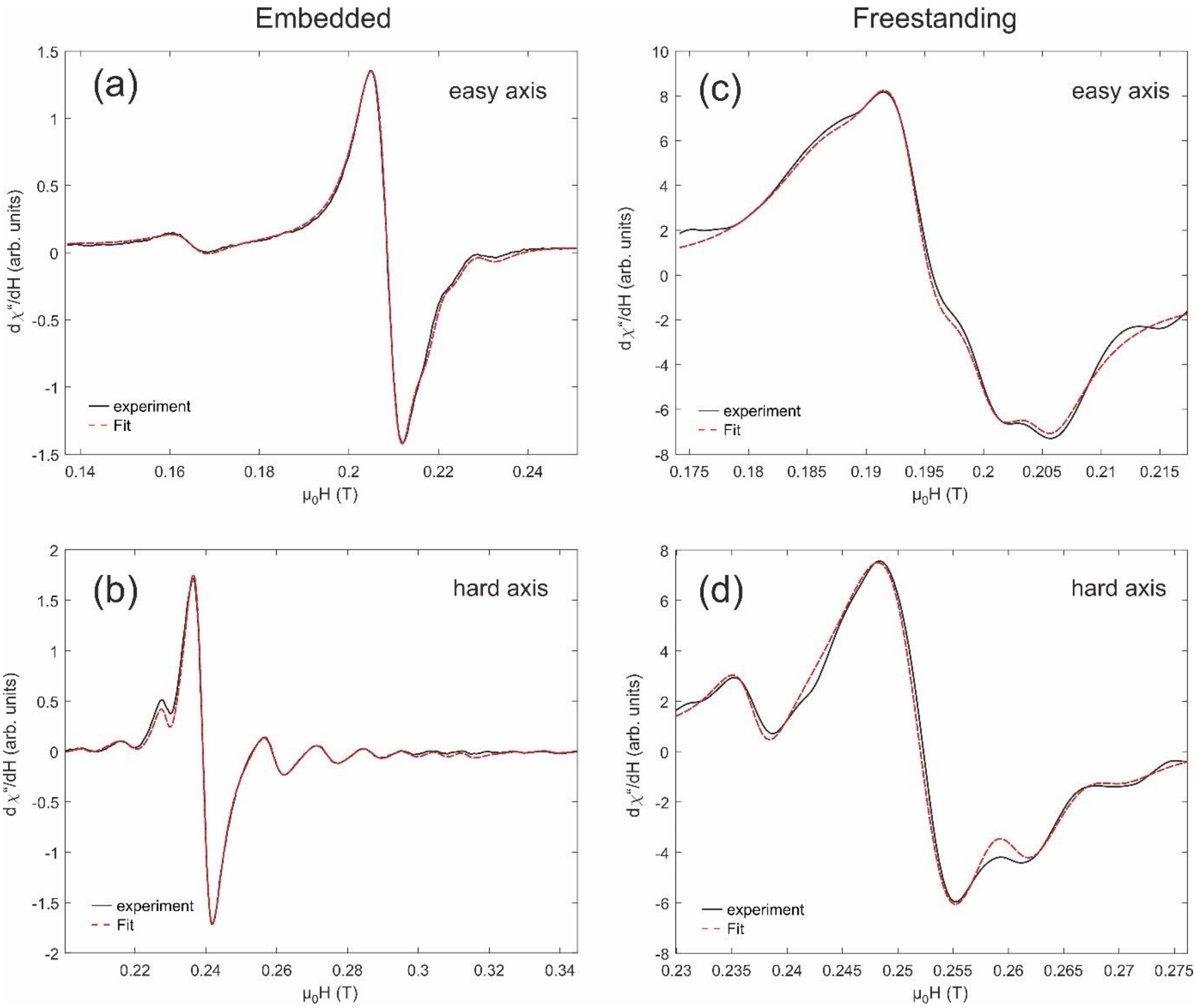

FIG S3. FMR spectra (black solid curves) showing the derivative of the absorption signal of (a,b) the embedded and (c,d) free standing strip with field along the easy axis of magnetization (a,c) and hard axis (b,d), respectively. Red dashed lines are multiple-line Lorentzian fits.

Figure S3 shows the FMR spectra for the embedded and freestanding strip in easy and hard axis geometry, respectively. The field modulation technique was used to improve the signal-to-noise ratio using lockin detection. Thus, the detected signal becomes the field-derivative of the FMR absorption line given by Eq. (S2):

$$\frac{\partial \chi''}{\partial H} = \frac{-16A\left(\frac{H-H_{\mathrm{res}}}{\frac{1}{2}\Delta H_{\mathrm{pp}}}\right)}{\left(3+\left(\frac{H-H_{\mathrm{res}}}{\frac{1}{2}\Delta H_{\mathrm{pp}}}\right)^2\right)^2} + \mathrm{Offset} + \mathrm{Slope}\cdot(H-H_{\mathrm{res}}) \tag{2}$$

Here, A is the FMR amplitude (intensity), offset is the y-axis offset, Hres is the resonance field position, and ΔHpp is the peak-to-peak linewidth of the signal. Finally, a sum of

Lorentzian derivatives with a common background (offset and linear slope) is applied to fit the multiple resonances of the spectra.

References:

S1. K. Wagner, L. Körber, S. Stienen, J. Lindner, M. Farle, A. Kákay, IEEE Magn. Lett, **12**, 6100205 (2021).

S2. M. O. Liedke, M. Körner, K. Lenz, M. Fritzsche, M. Ranjan, A. Keller, E. Cizmar, S.A. Zvyagin, S. Facsko, K. Potzger, J. Lindner, J. Fassbender, Phys. Rev. B, **87**, 024424, (2013)
